# Willingness to share information for energy efficiency: exploring differences and drivers across the Nordic countries

**DOI:** 10.1186/s13705-022-00363-3

**Published:** 2022-09-16

**Authors:** Joseph Anthony L. Reyes

**Affiliations:** International Institute for Industrial Environmental Economics (IIIEE), Lund University, P.O. Box 196, SE_221 00 Lund, Sweden

**Keywords:** Energy efficiency, Eurobarometer, Climate change perception, Behaviors, Willingness to share personal information

## Abstract

**Background:**

There is growing attention and policy debate about the sharing of personal information that the modernization of electricity grids requires. This is particularly important for big data management in smart grids that needs access to data generated and sent through devices such as smart meters. Using the Nordic Countries as a case study, this study investigates the willingness of people to share personal information for energy efficiency. The study builds upon data from the Eurobarometer survey and binary logistic regressions.

**Results:**

Nordic countries exhibit a higher willingness to share personal information compared to the rest of the EU countries. However, despite high levels of concern for climate change and other pro-environmental attitudes found overall among Europeans, the willingness to share personal information is not as prevalent and is still mainly shaped by socio-demographic features such as gender and age. Key predictors also included climate change perception and congruence of citizen engagement with environmentally friendly behaviors. Several contextual and market-specific issues framing these findings are discussed (e.g., trust, energy use).

**Conclusions:**

Even when high levels of pro-environmental attitudes in certain countries are found, let alone the Nordics, this does not mean people are willing to share personal information that would support pro-environmental energy efficiency behaviors and policies.

## Background

Climate change and sustainability goals can only be reached if smarter use of energy is widely applied [1–4]. There is increasing interest worldwide on matters of energy security, environmental pollution and climate change [5, 6]. The modernization of energy systems to smart grids requires sharing personal information [7–9], as such this article address the important need to for smart technologies such as the smart meter to be installed, and to gain and maintain access to the smart meter data from individual energy consumers (such as their energy usage amount, time, patterns, source, etc.) as this data will contribute to the overall development and efficiency of a smart grid.

Towards addressing the issues of sustainability with current development paths, consumer behaviors, and policy decisions, it is considered that constraints on energy availability and climate change are human-made problems. Correspondingly, mitigation of these challenges entail changes to behaviors, policies and institutional arrangements [10], with citizens in Western democracies playing a crucial role as they consume a larger amount of energy relative to the rest of the world [11, 12]. Homes and buildings have become more dependent on electricity, claiming a growing share of the budgets of households and firms, and raises the impetus for smarter usage of electricity [13]. For smart technologies, impediments nonetheless still abound as there had been observed delays in rollouts of member states for smart meters in recent years [14, 15]. The detrimental effect of the Covid-19 pandemic are also yet to be taken into account—with its implications on myriad issues such as residential electricity demand fluctuations due to Covid-19 restrictions [16], and on social–psychological factors with residents’ acceptance of and willingness to pay for home energy management systems [17]. Based on seeming tensions within the trends of current consumption and the (un)willingness to share personal information for energy efficiency is where the current paper situates itself by analyzing European citizens, with the Nordic countries taken as a case study. Specifically for the case of energy in the Nordics and as also observed in other contexts, the installation and maintenance of smart grid technology may be perceived as entailing immediate inconvenience and personal risks in terms of loss of comfort, violation of privacy, complex installation [7, 18–20].

As elaboration to better understand why such work is relevant and needed, the first and foremost reason for addressing them together is that Nordic countries are considered as being close to each other in terms of geography, culture, and socio-economic structures [21], as well as having similar climate, building standards and sharing a common energy exchange [22, 23]. With their proximity, all Nordic countries (with the exception of Iceland) can trade within the Nordic electricity exchange (Nord Pool) wherein the price of electricity is determined mostly by non-fossil-based power sources, price levels of fuels, and governmental policy and incentives. Major changes in the electricity markets in the Nordic countries have been observed as moving towards international markets, along with electricity users able to choose their electricity suppliers [24]. Particularly in the Nordic countries, household customers are becoming more aware of electricity consumption due to growing electricity price volatility in the market [22]. Smart technologies utilizing shared consumption information allow integration of energy infrastructures, services, renewables share and more efficient energy distribution, storage and utilization [24, 25]. It must however still be acknowledged that consumers or energy systems cannot be readily assumed to be identical across the world, that there can still be various levels of differences among countries [21]. Even the five Nordic countries are not uniform, with each continuing to see different market features emerge [26]. Nevertheless, given the aforementioned aspects and purported similarity in a global perspective [23]—it would be sensible in understanding the Nordics together in this study due to their relative closeness geographically, culturally, socio-economic structures [21] with respect to their energy practices.

Second, another particular point of interest for Nordic countries is that their societies are described as possessing high level of citizen's trust in public and private institutions [27]. Trust which can be understood as a sense of confidence and expectations from institutions and infrastructures [28], as well as trusting that someone is doing something, for the best of society [24]. People across Europe are considerably divided in their support of climate policies, with highest levels of support found in the Nordic countries. At the individual level trust in public institutions is considered as an important driver of a newly emerging eco-social divide, as well as traditional left–right political divides [29, 30]. High levels of social and institutional trust found in Scandinavia are considered to be a cooperative advantage when citizens consent to the sharing of personal information deposited in databases [31], as well as the acceptance of digital services [27]. The success of organizations in Europe handling these types of services are determined by the perception of how trustworthy they are in handling data [32]. Conversely, the lack of trust has been attribute as a barrier for consumers’ willingness to share information, acceptance of digital services such as e-commerce and internet banking practices, as well as new technical, regulatory and market solutions related to energy [28, 33–35]. With the development of information technology and big data, at present there is much yet to be understood about the challenges that extraction, commodification, and control of personal information presents to trust-based societies such as the Nordics [27].

Moreover, the data generated and sent through smart such as smart metering devices, could potentially have collateral implications for the end-users, these thus necessitate the building trust among people to overcome consumer resistance and successfully engage them in energy project, as well, as addressing the risks to highly trusting societies such as the Nordic countries wherein trust can be undermined and weakened [27, 34].

Third, with the current progress and enormous potential for developing new smart grid solutions and optimizing current grids in Nordic countries [36, 37], the need for further understanding of the Nordic region with its aforementioned barriers together with corollary issues such as legal aspects related to privacy and misuse of consumer data [37] becomes even more profound. Despite the seeming challenges, the Nordics for instance had been lauded for having established one of the first common electricity markets even when its countries had different electricity mixes and varying support schemes for renewables [36]. Two large advanced metering infrastructure platforms in the Nordic countries had been deployed in the last decade by a European multinational energy company (Schneider Electric) for the largest utilities in Norway (Fortum), Finland (Fortum), and Sweden (Vatenfall) [38]. In more recent years, on a national level, countries such as Sweden and Denmark have been prompting cities and communities to undertake their own path to sustainability and adopting myriad approaches towards GHG reductions [39]. For example in Sweden, progress had been made in smart buildings, zero-energy and plus-energy houses with governmental and private initiatives designed utilizing shared consumer information—with the aim of bringing about incremental and radical changes in terms of the building sector and deep renovations [37, 39, 40]. It had been however observed that though there are substantial efforts in reducing energy usage through technological developments and building practice in the Nordic countries, substantial gaps still remain between estimated and measured energy usage, with the major causes being household practices and consumer behavior [23]—presenting the impetus for further investigation of pro-environmental behaviors and social norms on energy reduction and climate change action [41, 42]. Previous studies discuss the potential in Europe for big data in improving public services and energy systems with entailing transformations within its societies [27, 43], while other research using Eurobarometer surveys have also investigated climate change oriented behaviors, such as 'installation of home equipment to control and reduce energy consumption (e.g., smart meter)' [7, 44, 45]. Reports that also refer to Eurobarometer results regarding the impact of digitalization on daily lives of Europeans shows support for sustainability and sharing personal information to improve public services, advocating the benefits that digital technologies offer and calling for wider engagement of citizens and ensuring opportunities to become available across European nations [46–49].

However, there is a dearth in studies utilizing recently available Eurobarometer data that focus on the willingness of people to share their personal information that would enable smart technologies towards the improvement of public services pertaining to energy efficiency. This article contributes to this gap in the literature with the new model employed for its analysis and in highlighting the important role of consumers, particularly those in the Nordic region, and the significant determinants of their willingness.

Hence, within these aspects of closeness, trust and expectations in society and institutions, and energy efficiency efforts within the region for climate solutions, the analysis of this study sets forth in specifically addressing the relevance of willingness of people for sharing information to climate change and other substantive factors. The discussion further considers overall progress made by the Nordic countries in terms of energy use reduction and efficiency, as well as carbon emissions intensity. The significance and novelty is in providing a meaningful addition to the extant literature on energy transitions and offering new perspectives and approaches that have not yet been done in other studies on consumer attitudes and behaviors to discuss potential implications to socio-technical, political and techno-economic mechanisms that potentially drive energy transitions and feasible climate actions.

The main objective of this study is to investigate the determinants of willingness to share information. It uses data primarily from the recent Eurobarometer survey (Wave 92.4) on “Attitudes of European citizens towards the Environment”, conducted in 28 European Union (EU) countries, focusing particularly on the Nordic region (Denmark, Finland, and Sweden). As presented later in the paper, another recent Eurobarometer survey (Wave 91.3) also includes a question on whether respondents 'installed equipment in their home to control and reduce their energy consumption (e.g., smart meter)'. The results from various European countries are included in the discussion, although the survey question may still leave room for interpretation as it is not solely about the smart meter, and could depend more on availability of the equipment and/or service—instead of the willingness of the consumer to install such smart meter technology and securely send their personal information securely to the smart grid for the purpose of energy efficiency which is the main concern of this study. Understanding the ‘willingness of people to share personal information securely for the purpose of improving energy efficiency of public services’ is the focus of the paper. Although smart meters are not explicitly singled out by the questions in the Eurobarometer survey (wave 92.4) which presents a challenge semantically, nevertheless smart meters play a crucial role in enabling individual consumers’ energy information to be shared, transmitted and be used for developing energy efficient smart grids. The use of public services in the Eurobarometer question can also be interpreted as provision by energy utilities to the general populace that can become more efficient with insights gained from smart data from the consumers. As such, the analyses investigates the following research questions:

1. What is the relationship of willingness to share personal information for energy efficiency and climate change perception?
2. What are the other substantive factors that significantly predict peoples’ preferences to share (or not) information?

Further discussions and insights are offered related to transitioning into a smart energy system on energy use reduction, challenges, and relevant consideration of emissions from a consumption-based perspective, encompassing energy efficiency in the residential end-use sector and relevant policy aspects to the low-carbon energy transition via empirical exploration of energy consumption, tackling the Nordics among EU countries.

## Methods

### Sample

Even with the abundance of data available on the subject of energy, surprisingly at present there is still a gap of knowledge for studies that focus on the consumer attitudes and behaviors of the Nordic region [42] who are the heaviest users but at the same time are also the forerunners of advancing energy technologies [26, 50]. While it must be acknowledged that many factors can determine energy use such as climatic conditions, industrial base, population growth, end-use technology, quality of building stock, income and energy pricing [42]. This said, though average energy consumption per capita is still higher across Nordic countries, it is also observed that Nordic countries are leading the deployment and growth of renewable energy shares in primary energy supply and energy efficiency [26, 50], such that energy efficiency and decarbonization pushed down their CO_2_ emissions below the European average from 2012 onwards [51]. Nordic countries have progressively reduced the role of fossil fuels in their respective buildings sectors as well as having overall marginal improvements in energy efficiency of buildings over the recent decades, its urban areas are expected to grow at twice the rate of previous decades. This creates an opportunity for transitioning into efficient low-carbon systems [51]. There is impetus for a deeper understanding of Nordic countries, as they are often touted as exemplars in sustainability among various fields such as energy and technological innovation [36, 42, 50], but at the same time the Nordics are heavy power consumers (50 GJ/capita), having higher electricity demand in all end-use sectors in comparison to Europe (20 GJ/capita) and the rest of the world (10 GJ/capita) [51]. In 2020, world electricity consumption per capita (MWh/capita) was 3.3, OECD total average was 7.5, Europe average was 5.5, the United States was 12.3, and People's Republic of China was 5.1, whereas for individual Nordic countries was at 5.7 for Denmark, 14.7 for Finland, 51.0 for Iceland, 23.3 for Norway, and 12.3 for Sweden [52].

The methodology of this paper is based on a quantitative empirical approach, utilizing data from the Eurobarometer survey “Attitudes of European citizens towards the Environment”, Special Eurobarometer 501 Wave EB92.4 with fieldwork conducted in 2019 and published on March 2020. The surveys were carried out by Kantar in the EU member states employing a questionnaire for computer assisted face-to-face interview with aged 15 above, from different social and demographic categories interviewed at home in the native language. A multi-stage, random (probability) basic sample design was applied in all states. In each country, a number of sampling points was drawn with probability proportional to population size (for a total coverage of the member state) and to population density.^1^ The current Eurobarometer dataset has 27,498 respondents from the 28 EU member states (UK was included) at the time of fieldwork, having a sample size of about 1,000 people in most countries while having about 500 people for smaller countries, with an almost equal number of respondents of each gender. For the purposes of the current case study, the focused analysis for the Nordics is restricted to the subsamples of the three countries: Denmark (1,026), Finland (1,007), and Sweden (1,012) collected in the period between 6 and 19 December 2019 containing 3,045 total observations.

### Variables

The main variable analyzed is the willingness of people to share their personal information securely to improve energy efficiency. Different variables are used to develop and frame the analysis, the selected items for the model are similar to those routinely used in environmental studies with the Eurobarometer datasets [10, 43, 44, 53–63]. As a novel aspect and contribution of the present study is that the latest Eurobarometer dataset item on willingness to share personal information for energy efficiency has yet to be operationalized at the time of this writing, particularly the question: “Public services could be improved if people shared some of their personal information. For what purposes would you be willing to share some of your personal information securely?”, with Respondents’ answering “To improve energy efficiency”[64]. Thusly other relevant variables were specifically chosen and excluded from the available items of the recent Eurobarometer dataset to explain the outcome, guided by previous studies that employed corresponding variables in their models addressing willingness [17, 65, 66] relevant to smart technology, stated preferences and environmentally significant behavior of citizens [53, 58, 59, 67].

This study employs indicators such as climate change concern, environmental attitudes, and behaviors: comprising five single-item variables and two composite indexes, with the model also including socio-demographic variables: gender, age, social class, education, having children, life satisfaction, marital status, political orientation, and community type [10, 29, 30, 57, 61, 66]. In the order of importance for the purpose of this study, the Special Eurobarometer 501 Wave items information are elaborated as follows in Table 1, with the description, coding, and relevant research that the variables were adapted from.

**Table 1:** Summary table of main variables

Main variables	Description	Original items	Coding	References to studies
*Dependent variable*				
Willingness	Willingness to share personal information securely to improve energy efficiency	qc6.3	1 (Yes), 0 (No)	Godoy et al. [27]; Lewandowsky et al. [43]
*Independent variables*				
Climate change perception	Additive index from two items measuring how serious a problem climate change is at this moment: In the country 1 (Not at all a serious problem) to 10 (An extremely serious problem); In the EU 1 (Not at all a serious problem) to 10 (An extremely serious problem)	qa2_1, qa2_2	2 (Lowest) to 20 (Highest)	Budžytė and Balžekienė [55]; García-Muñiz and Vicente, [66]; Ortega Egea and García de Frutos [44]; Sohlberg [73]; Wicker and Becken [10]
Environment protection importance	Personal importance of protecting the environment	qa1	1 (very/fairly important to the respondent personally), 0 (otherwise)	García-Muñiz and Vicente [66]; Golob and Kronegger [67]; Meyer [59]; Ott and Soretz [74]
Environmental behaviors	Sum score from items on environmental actions done by the respondent in the past six month, 1 (Yes) 0 (No): Chose environmentally friendly way of travelling; avoided over-packaged products; avoided single-use plastic; separated waste; reduced water consumption; reduced energy consumption; bought products marked with environmental label; bought local products; used car less; joined demonstration, workshop, collective activity; changed diet to more sustainable food; spoke to others about environmental issues; bought second-hand instead of new; repaired instead of replacing a product	qa6.1, qa6.2, qa6.3, qa6.4, qa6.5, qa6.6, qa6.7, qa6.8, qa6.9, qa6.10, qa6.11,qa6.12, qa6.13, qa6.14	0 to 14	García-Muñiz and Vicente [66]; Golob and Kronegger [67]; Liobikienė and Minelgaitė [60]; Orviska et al. [62]; Wicker and Becken [10]
Social norm	Belief that citizens are doing enough in their responsibility to protect the environment	qa9_2	1 (Yes), 0 (No)	Golob and Kronegger [67]; Gómez-Román et al. [56]; McCright et al. [57]; Ott and Soretz [74]; Urban and Kaiser [53]
Local infrastructure	Item measuring trust in the local authorities that the respondent's village/town/city is fulfilling its duty in protecting the environment, also considered as a proxy for the availability of green infrastructures at the local level	qa9_3	1 (Yes), 0 (No)	Pyrko and Darby [24]; Silvi and Rosa [61]; Urban and Kaiser [53]
Consumption change efficacy	Belief that changing the way we consume is one of most effective ways of tackling environmental problems	qa10.12	1 (Yes), 0 (No)	Connelly [63]; García-Muñiz and Vicente [66]; Novikovienė and Navickaitė-Sakalauskienė [75]

### Model specification and tests

For studies that have utilized variables from environmental surveys [18, 35, 68], tackling items such as the willingness to share personal information which assumes a binary or dichotomous value entail analyses with logistic regression or bivariate probit models [10, 59, 61, 66]. This paper adopts an approach utilizing logistic regression similar to established research on environmental attitudes and behaviors with Eurobarometer surveys relevant to energy [10, 44]. As an econometric model developed for a dichotomous categorical dependent variable more apt than linear models such as the ordinary least squares estimator, herein the binomial logistic regression formula takes the form [69]:

1 $${\text{Ln}}\left[ {\frac{{\pi (y)}}{{\{ 1 - \pi (y)\} }}} \right] = \beta 0 + \beta 1 \times 1 + \beta 2 \times 2 + \cdots + \beta {\text{nxn}} + \varepsilon$$

where Ln = natural logarithm, *π*(*y*) = probability of observing the outcome variable (share or not share information) equals one instead of zero, *β*0…*β*n = regression coefficients, × 1…xn = intrinsic and/or extrinsic explanatory variables, *ε* = error term. To determine the inclusion and retention of variables for the regression model, 'several automated statistical procedures are available that allow forward, backward, and stepwise selection of variables, with several user-modifiable criterion for variable selection' [70]. These methods are touted to assist researchers in generating and screening hypotheses, however careful consideration must be made in deciding relevant variables for the model, as the inclusion or removal of predictors is based entirely on statistical criteria [69, 71]. Thus, it is crucial that decisions refer also to the theoretical literature available, with the initial model based upon previous research that includes meaningful substantial and demographic variables [70–72].

For this study, the binary logistic regression method was conducted using SPSS version 25 program for the analyses with the “ENTER” simultaneous entry procedure for variables [18, 60, 76] applied to the pooled Nordic sample and individually for Denmark, Finland, and Sweden. To address a potential limitation of the model for the presence of some correlations among the regressors, checks for collinearity were carried out and did not reveal dependencies among the explanatory variables—with pairwise correlations indicating low correlations and computed Variance Inflation Factors (VIF) below five suggesting that multicollinearity not being an issue [61, 69, 77]. The Hosmer–Lemeshow test result for the model was found to be not significant indicating that it is a good fit [70, 78]. The analysis also involves the application of descriptive statistics, nonparametric tests for independent samples and post hoc tests [79, 80] to establish differences in the characteristics between those respondents who are willing and unwilling to share personal information for energy efficiency.

## Results

For the main variable of the study, among the Nordic countries, Denmark (45.1%) had the most respondents stating that they are 'willing to share personal information securely to improve energy efficiency’; followed by Sweden (41.1%); and then Finland (24.6%) being the least willing. All three Nordic countries were above the percent willing of the non-Nordic EU countries (21.9%) and Pooled 28-EU country (23.5%) European Barometer sample (see Table 2). Results from chi square test show a statistically significant relationship (X^2^_3_ = 478.52, *p* = 0.000) between willingness to share personal information and country sample (Denmark, Finland, Sweden, Other non-Nordic EU countries). Post hoc was performed using Bonferroni correction that adjusts for the family-wise error rate [35, 81] indicating significant differences between categories in pairwise tests, wherein for the set of tests associated with those willing to share personal information for energy efficiency, the proportions of respondents in Denmark or Sweden are greater than the proportions of respondents in Finland or other non-Nordic EU countries. Whereas the tests associated with those who are unwilling to share information show that Finland and other non-Nordic EU countries had a significantly higher proportion than Denmark or Sweden.

**Table 2:** Descriptives for main variables

Main variables	Nordic pooled sampled		Other 25 non-Nordic EU countries		Denmark		Finland		Sweden	
	Mean	S.D	Mean	S.D	Mean	S.D	Mean	S.D	Mean	S.D
Dependent variable										
Willingness	0.37	0.48	0.22	0.41	0.45	0.50	0.25	0.43	0.41	0.49
Independent variables										
Climate change perception	15.18	3.98	15.47	4.12	15.47	3.94	14.59	3.89	15.47	4.06
Environment protection importance	0.97	0.18	0.94	0.25	0.96	0.18	0.95	0.22	0.99	0.11
Environmental behaviors	5.82	3.02	3.99	2.62	5.69	3.08	5.04	2.77	6.72	2.97
Social norm	0.35	0.48	0.32	0.47	0.41	0.49	0.34	0.48	0.31	0.46
Local infrastructure	0.52	0.50	0.42	0.49	0.58	0.49	0.54	0.50	0.43	0.49
Consumption change efficacy	0.41	0.49	0.31	0.46	0.38	0.49	0.47	0.50	0.37	0.48

Source: the author’s compilation from the ZA7602: Eurobarometer 92.4 dataset [64]

The model R^2^ values for the Nordic pooled and individual samples from Denmark, Finland and Sweden, were 0.163, 0.182, 0.153, and 0.123, respectively. Overall, these are comparable to environmental behaviors and energy efficiency studies which utilized the adopted methods and variables [18, 20, 35, 54, 68] for the analysis and also to those that had items similar to the recent Eurobarometer dataset, with model R^2^ values ranging from 0.075 to 0.263 [10, 61]. It should be noted that given the modest sizes of the statistically significant coefficients in Table 3, it is not surprising that, taken together the entire set of 16 predictor variables entered for the model does not account for much variance. This suggests that there may be a large amount of random variance in the respondents’ choices or the unavailability of important predictor variables from the model [18]. It must however be considered that even for other datasets from Gallup Polls, World Values Surveys, and European Values Surveys relatively low R^2^ values had been found to be typical among environmental survey studies [68, 79].

**Table 3:** Summary of regression results for willingness to share personal information securely for energy efficiency

	Nordic pooled sampled			Other 25 Non-Nordic EU countries			Denmark			Finland			Sweden		
	*β*	*p *	OR	*β*	*p *	OR	*β*	*p *	O	*β*	*p *	OR	*β*	*p*	OR
Climate change perception	**0.049**	**0.000**	**1.050**	**0.022**	**0.000**	**1.023**	**0.046**	**0.025**	**1.047**	0.029	0.299	1.029	**0.060**	**0.003**	**1.062**
Environment protection importance	0.204	0.494	1.226	**0.345**	**0.001**	**1.413**	0.766	0.125	2.151	0.172	0.724	1.188	− 0.804	0.222	0.447
Environmental behaviors	**0.126**	**0.000**	**1.134**	**0.116**	**0.000**	**1.123**	**0.111**	**0.000**	**1.118**	**0.159**	**0.000**	**1.172**	**0.120**	**0.000**	**1.128**
Social norm	− 0.169	0.087	0.844	0.041	0.380	1.042	− 0.235	0.149	0.790	− 0.222	0.270	0.801	− 0.089	0.596	0.915
Local infrastructure	− 0.017	0.858	0.983	− 0.064	0.156	0.938	− 0.140	0.384	0.870	0.164	0.380	1.178	− 0.034	0.828	0.966
Consumption change efficacy	− 0.077	0.374	0.926	0.020	0.611	1.020	− 0.183	0.216	0.833	− 0.017	0.920	0.983	− 0.031	0.831	0.970
Gender (Ref.: Man)															
Woman	**− 0.513**	**0.000**	**0.599**	**− 0.231**	**0.000**	**0.794**	**− 0.502**	**0.001**	**0.605**	**− 0.603**	**0.001**	**0.547**	**− 0.429**	**0.004**	**0.651**
Age	**− 0.012**	**0.000**	**0.989**	**− 0.008**	**0.000**	**0.992**	**− 0.012**	**0.036**	**0.988**	− 0.003	0.606	0.997	**− 0.015**	**0.003**	**0.985**
Social class (Ref.:Working, lower-middle class)		0.003			0.252			0.281			0.274			0.016	
Middle class	**0.327**	**0.003**	**1.386**	0.068	0.099	1.071	0.073	0.705	1.076	0.324	0.109	1.383	**0.533**	**0.007**	**1.704**
Upper-middle, higher	**0.464**	**0.001**	**1.590**	0.068	0.381	1.070	0.348	0.161	1.417	0.204	0.476	1.226	**0.631**	**0.010**	**1.879**
Education	0.007	0.711	1.007	**0.050**	**0.000**	**1.051**	0.002	0.952	1.002	− 0.005	0.903	0.995	0.029	0.441	1.030
Children (Ref.: No children)															
Has children	0.187	0.106	1.205	0.019	0.641	1.019	0.124	0.522	1.132	0.367	0.119	1.443	0.214	0.275	1.238
Life satisfaction (Ref.: Else)															
Very satisfied	− 0.091	0.323	0.913	**0.148**	**0.001**	**1.159**	− 0.069	0.679	0.933	0.031	0.866	1.031	− 0.157	0.283	0.855
Marital status (Ref.: Unmarried)		0.675			0.103			0.392			0.029			0.141	
Married/re-married/single with partner	− 0.034	0.788	0.967	**0.122**	**0.036**	**1.129**	− 0.327	0.134	0.721	− 0.387	0.150	0.679	0.345	0.078	1.412
Divorced or separated	− 0.086	0.617	0.918	0.027	0.761	1.027	− 0.280	0.388	0.756	**− 0.810**	**0.023**	**0.445**	0.489	0.061	1.631
Widowed	− 0.225	0.255	0.799	0.043	0.653	1.044	− 0.514	0.122	0.598	**− 1.097**	**0.009**	**0.334**	**0.634**	**0.050**	**1.886**
Political orientation (Ref.: Left)		0.707			0.000			0.332			0.943			0.948	
Center	0.013	0.902	1.013	**− 0.178**	**0.000**	**0.837**	− 0.055	0.743	0.946	0.059	0.786	1.061	0.057	0.748	1.058
Right	− 0.075	0.507	0.928	− 0.073	0.136	0.929	− 0.268	0.151	0.765	0.081	0.742	1.084	0.036	0.845	1.037
Community type (Ref.: Rural area or village)		0.000			0.075			0.000			0.008			0.285	
Small or middle-sized town	− 0.053	0.635	0.948	− 0.025	0.583	0.976	0.058	0.758	1.060	0.000	0.999	1.000	− 0.340	0.124	0.712
Large town	**0.393**	**0.002**	**1.481**	0.076	0.106	1.079	**0.787**	**0.000**	**2.197**	**0.677**	**0.006**	**1.969**	− 0.215	0.345	0.807
Country dummies	Included			Included											
Constant	− 1.156	0.006		− 1.425	0.000		− 1.048	0.130		− 1.990	0.008		− 0.898	0.264	
Nagelkerke *R*^2^	0.163			0.110			0.182			0.153			0.123		

Figures in each column are unstandardized B coefficients. Significant results in bold. Source: ZA7602: Eurobarometer 92.4 dataset [64]. Layout adapted from Wicker & Becken [10]

Among the most interesting findings for the predictors included in the models are the following. Climate change perception was found to be significant for willingness to share personal information securely to improve energy efficiency in the Pooled Nordic sample, and individually in Denmark and Sweden, but not Finland. The pro-environmental behavior sum score index (Environmental Behaviors) was found to be consistent among samples as significant and contributed strongly as indicated by the Wald statistic. In other words, the more environmental actions that were done by the respondent in the past 6 months, the more likely they would be willing to share personal information for energy efficiency. However, the variables pertaining personal importance of environmental protection, belief in social norm, trust in local infrastructure, and consumption change efficacy were not found to be significant in the pooled Nordic sample or individual Nordic countries.

In regard to socio-demographics, gender of the respondent was found to be significant in all samples, and the coefficient which is the strongest contributor to the model as indicated by the Wald statistic. Thus, among the available variables utilized in the analyses, gender was the most substantial and consistent as a predictor, wherein it is indicated that women are less willing to share their personal information as compared to men. Older respondents were less willing to share their personal information with age found to be significant with an inverse relationship to willingness in the Pooled Nordic sample, Denmark, and Sweden, though not in Finland. For the Nordic pooled sample and Sweden, results for social class indicate that those who identified themselves as being in the middle, upper middle, or higher class were more likely to share personal information. For marital status, being divorced, separated or widowed in Finland meant being less likely to share information, whereas in Sweden being widowed indicated being more likely to share. Education, life satisfaction, and political orientation were not found to be significant in the Nordic samples. In regard to community type, significant results were found in the Pooled Nordic sample, Denmark, and Finland, though not in Sweden—indicating that those living in large towns or cities were more willing to share their personal information for energy efficiency as compared to those living in rural areas or villages.

In terms of similarities and differences between the Nordics and the rest of EU countries: gender was found to be consistently significant in the pooled and individual Nordic samples as well as the Other 25 Non-Nordic EU countries sample wherein women were less willing to share personal information for energy efficiency. The most notable differences were found with environmental protection importance, education, life satisfaction, and political orientation being significant predictors for willingness in the Other 25 Non-Nordic EU countries sample but not among people in the Nordics.

## Discussion

The discussion takes the findings and their implications as differences and drivers in terms of: the Nordic countries’ closeness pertaining culture, geography, and socio-economic structures; high levels of citizen trust in their institutions and society; and the region’s current progress and enormous potential for developing energy solutions.

First, Nordic countries are different from the rest of the EU countries in terms of higher willingness to share personal information. However, this does not entail that the Nordic countries are homogenous, as demonstrated by non-parametric test and the consistency of predictors and their significance in the regression models.

Second, trust in local authorities and institutions, as well as social norms pertaining to belief that or somebody in the city or someone else is doing something [24] and fulfilling their duty in protecting the environment does not necessarily translate to an individual in the Nordic countries or the EU being willing to share their personal information for energy efficiency. It was found that the variable for local infrastructure measuring whether the individual trusts the local authorities and believes that the city is fulfilling its duty in preserving the environment, which can also be considered as a proxy for the availability of green infrastructures at the local level enabling pro-environmental behavior [61], was not a significant predictor in the Nordic pooled sample, the individual Nordic countries, the pooled non-Nordic countries, nor the entire 28-country Eurobarometer sample. This was also the case for the dummy variable social norm which can be considered as representing respondents’ belief that citizens themselves were fulfilling their duty in preserving the environment. More importantly, findings reveal that it was actually those who already ‘walk the walk’ through engagement in other pro-environmental behaviors who were found to significantly be more likely willing to share their personal information for energy efficiency.

Third, the attitudes, behaviors, and contexts of individuals should be carefully taken into consideration as willingness does not automatically entail the same relationships with factors/determinants ascribed to traditional environmental values, behaviors, or support to policy instruments. Other indicators such as personal importance of environmental protection, belief in social norms of citizens in protecting the environment, trust in local authorities and infrastructure, as well as the efficacy of changes in consumption had not been found to be significant for willingness to share in the Nordic countries, though these have been found in some literature as significant predictors for pro-environmental behaviors, as well as whether information about the energy consumed about online services influences service usage [61, 66]. Though this can also be considered as similar to observations pertaining the Eurobarometer that green attitudes of Europeans do not necessarily always translate into environmentally friendly behavior and concrete actions [56], what is distinctive in this study is that results show congruence in pro-environmental behaviors increases the likelihood of willingness to share personal information for energy efficiency.

Furthermore, looking at the country-level with the results obtained indicating climate change perception and engagement in significant behaviors as significant predictors, the potential interaction between the two can be further considered [30].

**Fig. 1:**
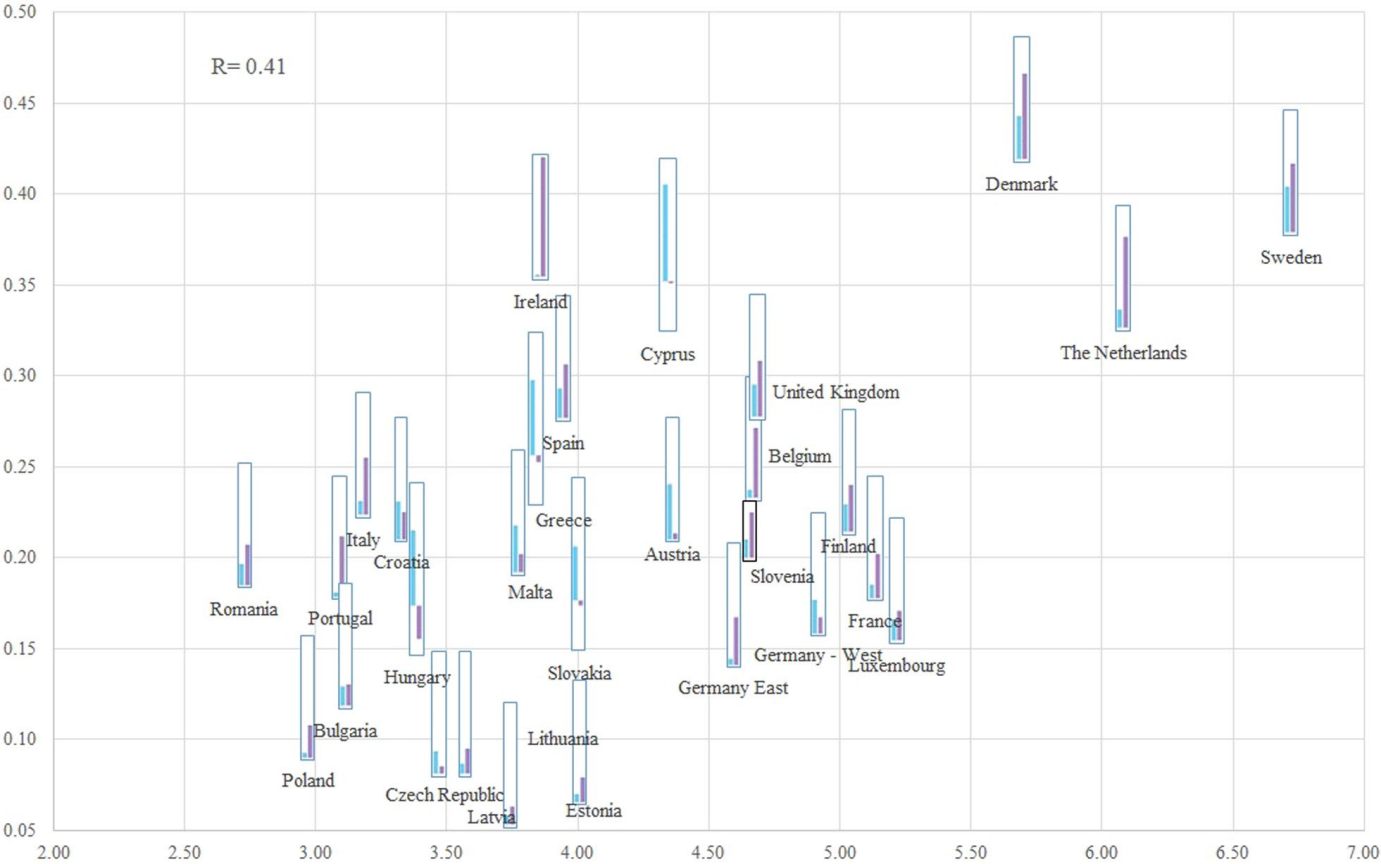
Willingness to share personal information for energy as a function of engagement in environmental behaviors. Cross-nationally, figure shows the relationship between climate change perception and willingness (vertical axis), plotted against engagement in environmental behaviors (horizontal axis). Two columns for each country show the intercepts and slopes from a bivariate, unconditional linear regression of willingness on climate change perception. *Note:* Figure adapted from Fairbrother (2019), with author using own compilation from the ZA7602: Eurobarometer 92.4 dataset [64]

To focus on this interaction, Fig. 1 shows the relationship between climate change perception and willingness to share personal information for energy efficiency within countries, presenting the mean national level of willingness (vertical axis) against mean national level of environmental behaviors engagement (horizontal axis). In turn, within each country point—bar plots represent the intercept (blue) and slope coefficients (violet) from a bivariate regression of willingness on climate change perception. These are estimated using ordinary least square (OLS) models and do not include any control variables, with intercepts showing the modeled willingness of people that place little importance to climate change [30]. Another way to put it, using the climate change perception variable re-centered to a minimum value of zero, intercepts from the individual country regressions show expected value of the outcome for respondents with the least possible perception to climate change issues.

Countries with higher levels of environmental behaviors and higher willingness (towards the upper-right of the graph) do not necessarily have notably higher intercepts, with correlation at 0.11 while 0.37 for correlation with willingness. Interestingly, results show observable clustering in terms of geopolitical and economic groupings of Eastern European/post-communist countries and Western European countries, with Denmark, Sweden, and Finland situated together at the upper-right quadrant.

Slope coefficients show the differences between people with low and high levels of climate change perception within each country. Cross-level interaction effect is visible in a seeming curvilinear relationship between the level of mean environmental behaviors (x axis) and the height of the violet bars: with heights of bars low at the center, while taller somewhat at the left and the right ends of the panel. The slope coefficients for the countries towards the upper-right of the graph are slightly higher, while the slope coefficients for the countries to the lower-left are lower. This suggests that the divide between those with low and high climate change perception is greatest in countries whose people engage in more environmental behaviors. The slope for perception varies substantially with the mean environmental behaviors in a country with correlation at 0.39. This increasing divide is also greater in countries with more willingness having a correlation of 0.55. A similar clustering of countries in terms of geopolitical and economic groupings as described above are also observed.

In more concrete terms, willingness to share information are highest in Denmark and Sweden, although Danes and Swedes are unremarkable in perceiving how serious a problem climate change is at this moment. If climate change perception is exceptionally high anywhere, it is in Bulgaria, where willingness to share information is very low. What also sets Denmark and Sweden apart are their steep slope coefficients on climate change perception and high levels in engagement of environmental behaviors.

Overall, findings of this study reveals that it is crucial to examine closely the citizens’ attitudes and their countries contextual factors particularly for the Nordic countries that are touted to be leaders in clean energy and efficiency [26, 50] in order to better appreciate their particular progress and further develop their potential. As such, in addition to understanding the individual citizens via Eurobarometer 501 Wave EB92.4, additional sources of relevant information can be utilized in contextualizing as well as examining the progress and trajectories of respective Nordic countries, particularly from a consumption-based perspective.

### Energy consumption in Europe and the Nordics

In situating the need and desire of people to share personal information for energy efficiency, it is important to understand how reduction in energy patterns of consumers had been driven in the recent past and its potential directions for the near future, it is useful to investigate measures implemented and examine the implications of such in addressing behavioral change towards further improving energy efficiency and climate change mitigation.

Member states of the EU have deployed various policy mechanisms to mitigate market failures associated with energy efficiency [82], among which the introduction of smart meters expected to ‘reduce CO2 emissions in the EU by 9% and annual household energy consumption by 10%’ [83]. Recent developments described in the December 2019 DG Energy European Commission Report "Benchmarking smart metering deployment in the EU-28" indicates that 34% of all household and SME metering points had been equipped with a smart meter as of 2018 (ca. 99 million smart meters), particularly with households electricity metering points equipped at 35% [14, 15]. The EU as of 2020 is reported to having the second highest smart meter penetration rate after North America [84]. With EU member states proceeding with their smart meter rollouts, it is expect that overall (in households and SMEs) 223 million smart meters will be installed by 2024 (corresponding to a 77% penetration rate), representing an aggregated investment of €38 billion, and by 2030, 266 million smart meters will be installed (corresponding to a 92% penetration rate), which would represent a total aggregated investment of €46 billion [14].

It must be noted that a central tenet of the measure for smart grid deployment is that energy consumption information provided through the smart meter will enable end-users to make wiser and, in principle, “rational” decisions about their energy service demands [82], intended to transforming consumers from ‘passive participants to active users and optimizers of their extended energy possibilities’ [13, 65]. Informational feedback has been used extensively as a tool to increase user knowledge and motivation [66, 85]. However, among substantial barriers for smart meter technology utilization in the EU relates to privacy, data security, safety concerns and reluctance to share information [4, 25, 28, 34, 35, 86, 87]. Issues include concerns about the use of data energy consumption by utilities and/or third parties, as well as fear of crimes such as break ins due to knowledge of when a consumer is at home or not [14, 88, 89].

In terms of pursuing targets such as these through smart meter opportunities, although Finland and Sweden have been front runners in the roll-out of smart meters in Europe, with Norway and Denmark closely following with their respective roll-outs—there are still at present factors that hamper efforts to accelerate energy efficiency investments such as asymmetric information, technological risks, lack of adequate capital, transaction costs, long payback periods and split incentives in rental situations among others [14, 51, 82, 90–92].

As also reflected in the results of a recent Eurobarometer survey (ZA7572) conducted in 28 member states in the EU, for the total average only 14.8% (4095 of 27,655 respondents) mentioned having installed equipment in their home to control and reduce energy consumption (e.g., smart meter) [93]. It was only in the Netherlands sample wherein there was a higher portion of the respondents who installed equipment (51.2%). The Nordics still overall had higher percentage of affirmative respondents as compared to the EU-28 total sample (Table 4), among which Denmark had the highest percentage (25.8%), followed by Finland (22.5%), and then Sweden (16.2%).

**Table 4:** Cross-tabulation of Eurobarometer survey item (qb6.8) on climate change action: energy saving equipment at home

Country	Not mentioned (%)	You have installed equipment in your home to control and reduce your energy consumption (e.g., smart meter) (%)
Netherlands	48.3	51.7
United Kingdom	69.3	30.7
Denmark	74.2	25.8
Luxembourg	74.3	25.7
Malta	74.6	25.4
Finland	77.5	22.5
France	79.7	20.3
Ireland	81.6	18.4
Spain	82.3	17.7
Austria	82.9	17.1
Sweden	83.8	16.2
Belgium	83.9	16.1
Latvia	84.1	15.9
West Germany	85.3	14.7
Slovenia	85.9	14.1
Estonia	88.7	11.3
Cyprus	89.3	10.7
Czech Republic	90.3	9.7
Poland	91.6	8.4
Hungary	91.9	8.1
East Germany	92.0	8.0
Italy	92.1	7.9
Slovakia	92.7	7.3
Lithuania	92.7	7.3
Romania	93.5	6.5
Portugal	93.8	6.2
Croatia	94.8	5.2
Bulgaria	96.8	3.2
Greece	97.5	2.5
Total % within sample (27,655)	85.2	14.8
Total count	23,560	4095

Source: The author’s compilation from the ZA7572: Eurobarometer 91.3 dataset [64]

**Fig. 2:**
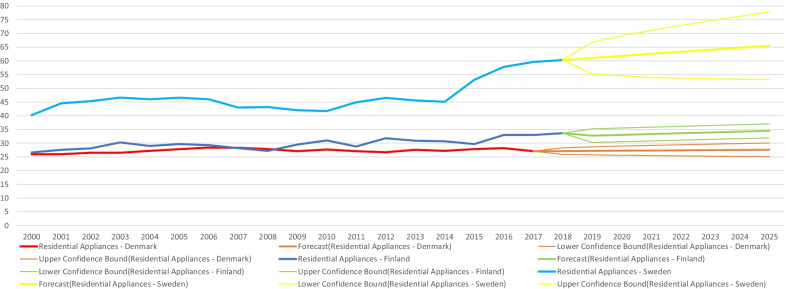
Residential appliances total consumption (in PJ) and forecasts for selected Nordic countries. *Note:* Data compiled by authors from IEA Energy Efficiency Indicators https://www.iea.org/subscribe-to-data-services/energy-efficiency-statistics, forecast at 95% confidence interval

**Fig. 3:**
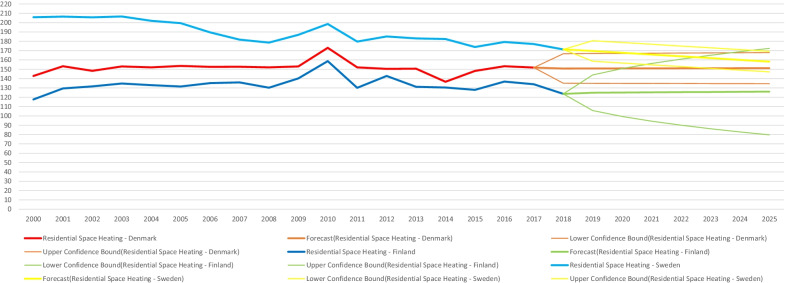
Residential space heating total consumption (in PJ) and forecasts for selected Nordic countries. *Note:* Data compiled by authors from IEA Energy Efficiency Indicators https://www.iea.org/subscribe-to-data-services/energy-efficiency-statistics, forecast at 95% confidence interval

**Fig. 4:**
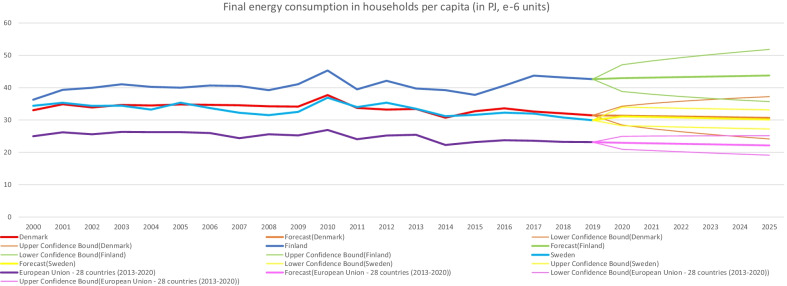
Final energy consumption in households per capita (in PJ) and forecasts. *Note:* Computed by authors with data from Eurostat database https://ec.europa.eu/eurostat/databrowser/product/page/SDG_07_20, forecast at 95% confidence interval

**Fig. 5:**
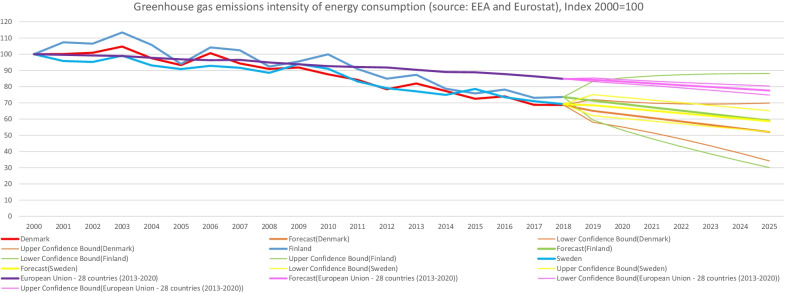
Greenhouse gas emissions intensity of energy consumption, Index 2000 = 100. *Note:* Data compiled by authors from Eurostat database https://ec.europa.eu/eurostat/databrowser/product/page/SDG_07_20, forecast at 95% confidence interval

The focus of this current study on sharing personal information for energy efficiency naturally relates to the particular case of energy use in the residential sector. As demonstrated in data from the IEA when considering Nordic countries experiencing increases in population and economic growth—for instance in total energy expenditure (in Petajoules) for residential appliances (Fig. 2) and space heating (Fig. 3) they still manage over time to keep relatively minimal increases—similarly for residential appliances per capita energy intensity (index 2000). Though being heavy power consumers compared to Europe and the world, the Nordics have notably consistent decreasing trends for Total Residential consumption by end-use per capita energy intensity [12]. When comparing with figures for the EU-28 from Eurostat and the EEA—final energy consumption in households per capita in Denmark, Finland and Sweden was higher (Fig. 4), notably at the same time overall decreasing greenhouse gas emissions intensity has been indicated in the data, with intensity among the three Nordic countries having been lower than the EU-28 since 2011 (Fig. 5).

**Fig. 6:**
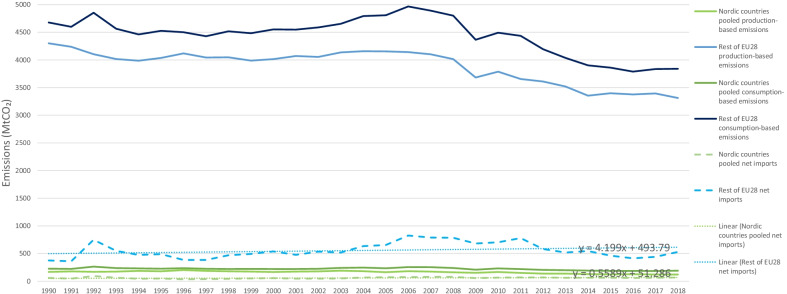
Production- and consumption-based CO_2_ emissions and resulting net imports. Data from Peters et al. 2011. Growth in emission transfers via international trade from 1990 to 2008, Proc. Natl. Acad. Sci. 108, 8903–8908; Friedlingstein et al. 2020. Earth Syst. Sci. Data. 12, 3269–3340; Global Carbon Atlas. Available at http://globalcarbonatlas.org/en/CO2-emissions

From a consumption-based perspective which is highly applicable to the residential sector, net imports of emissions being positive is worth careful consideration. Quantitative studies show that Scandinavian countries have high carbon emissions embodied in imports that satisfy domestic consumption [42, 77, 94]. Taking in the case of Nordic countries, the results indicate a negative emission trading balance (see Fig. 6). However, as a whole for the three Nordic countries included in this study, net imports of emissions have grown by merely about 14% since 1990, an average of about less than 0.5% per year. Figures do show some discrepancies among countries: net imports grew by 108% in Denmark, 5% in Sweden, and shrunk in Finland by − 6% for the period 1990–2015. Further, recent data for 2018 indicate that when compared to other industrialized countries, the pooled net imports of emissions of the three Nordic countries (67.5 MtCO_2_) are less than those of countries such as France (110.3 MtCO_2_) or Germany (106.2 MtCO_2_), and not even coming close to half of the net imports of the United Kingdom (160.0 MtCO_2_). Moreover, the increasing trend among the net imports of the three Nordic countries, is substantially lesser as compared to the increasing trend found for the rest of the EU 28 countries [95]. Nonetheless, in line with earlier observations [42, 77] that although there had been discernible progress in reducing production-based (or territorial) emissions, these recent trends still indicate that greater policy efforts are necessary to similarly further reduce consumption-based emissions and curtail resultant increasing net imports.

This analysis suggests that despite the higher relative levels of consumption in households per capita, overall there were still better improvements in patterns of energy use and decarbonization across Nordic countries, such as in terms of production-based and consumption-based emissions as compared to the rest of the EU 28 countries for the period 1990–2018.

With these development in mind within the already progressive societies such as those found in Nordic countries and the EU broadly, in terms of attitudes and infrastructures that drives low-carbon and energy efficiency, a deeper understanding of consumers would be paramount especially when concerning decisions needed for further reducing consumption of energy and increasing the willingness to share personal information to utilize smart technologies. As demonstrated together with the findings from recent Eurobarometer surveys [64, 93] covered in this study, there is consensus across countries regarding the severity of climate change and the necessity to take actions both individually and at the societal level [85].

This section offered a discussion of the relevant context and challenges in Nordic countries, as well highlighting the potential and critical role it can play towards energy efficiency considering the levels pro-environmental attitudes and behaviors, trust of its citizens, climate change perception, and levels of acceptance of energy saving equipment such as smart meters [1, 15, 27, 28, 34, 42, 84]. As discussed in this section on energy consumption in Europe and the Nordics, and touched upon in earlier sections of the paper, in addition to understanding the individual citizens' willingness to share personal information for the purpose of improving public services for energy efficiency which is described primarily in the Eurobarometer dataset of this study: in order to situate the need and desire of people for this willingness, it can be helpful to refer to various informational sources and important to understand how reduction in energy patterns of consumers had been driven in past years, at present and the potential directions in the near future. Such understanding of the congruence of individual sentiments and behaviors, along with the empirical aspects at the country levels would be useful in guiding efforts in terms of adequacy and scope for future energy efficiency and climate change mitigation strategies.

### Policy implications

Policy formulation and implementation could benefit by considering citizens’ approval of environmental policies and willingness to accept climate change policies and low-carbon innovations, through factors such as peoples’ attitudes, preferences for energy technologies and pro-environmental ‘behaviors that entail effort or inconvenience’ [96]. Low-carbon innovations such as those enabled by smart meter technology is considered as 'integral to energy transformation for climate change mitigation, and could serve as more efficient or produces less carbon than incumbent forms of energy production, distribution or use—but has limited consumer appeal' [97]. Moreover, beyond technology substitution, understanding the demand and willingness for energy transitions highlight the importance of social factors—also encompassing elements such as user practices, regulation, perceived costs, risks, benefits, industrial networks and infrastructure [98–100] and the significant determinants among peoples identified in this study should also then be taken into account in policy formulation and decision-making.

Particularly salient among the significant findings of this study is citizens’ engagement in environmental behaviors which remained consistent throughout the Nordic pooled sample and individual Nordic countries, as well as the other 25 EU member countries—in line with other research further demonstrating that those who were environmentally responsible tended to be have more environmentally friendly values [60, 62, 66] further highlighting the influence and importance of congruency in behaviors [10, 46]. It is, however, acknowledged that a limitation of the present study is that the Eurobarometer dataset (Wave 92.4) does not have questions on specific smart technologies, and it is hoped that future Eurobarometer waves and other studies conduct research employs additional survey items pertaining particular technologies and consumer behaviors associated. As can be similarly noted from a related study on environmentally oriented anticonsumption and consumption using five Eurobarometer surveys from 2009 to 2017 [44], a deeper understanding of climate change oriented behaviors could be beneficial with further details on particular smart technologies given recent advancements and applications.

Another key finding of the study was women being less likely to be willing to share personal information for energy efficiency—which goes against commonly held belief and findings in other environmental consumption studies that purport women as being more ecologically conscious and make more environmentally friendly decisions than men do [20, 44, 59], but at the same time is in line with studies that found gendered differences in the energy sector such as women having lower risk tolerance and lower trust in technology [101] as well as insecurities related to personal information such as those in the sharing economy and how consumers perception of the roles of information-based services varies by gender [102, 103]. Given these new insights together with the increasing dual role of people in exercising both political and market power [104] in the Nordics, EU and beyond, who are becoming more concerned across sectors due to pressures arising from the environment [105–107]—with international institutions, standards, or examples from other countries further encouraging states to adopt new energy policies [108].

## Conclusions

This paper presented evidence on willingness to share personal information for energy efficiency, with Nordic countries exhibiting a higher willingness compare to the rest of the EU countries. Despite high levels of concern for climate change and other pro-environmental attitudes found overall among Europeans, the willingness to share personal information was not as prevalent and is still mainly shaped by socio-demographic features such as gender and age. Key predictors also included climate change perception and congruence of citizen engagement with environmentally friendly behaviors. Empirical analyses also showed similar patterns in terms of overall low rates of individuals having installed equipment in their homes to control and reduce energy consumption. Higher final energy consumption in households per capita was found across Nordic countries as compared to the EU as a whole, however greenhouse gas emissions intensity of energy consumption had been steadily lower than the EU average since 2011. Better relative improvements are also found across Nordic countries in terms of production-based and consumption-based emissions as compared to the rest of the EU 28 countries further demonstrating progress and potential for developing innovative energy solutions.

The findings have timely implications which can aid policy and decision-makers in Europe and the Nordics in particular, towards capitalizing on the potential for low-carbon energy innovations and transitions, highlighting the importance of getting people to become more willing to share their personal information for energy efficiency. In addition to carefully considering the resounding voices of climate change concern and socio-demographic preferences—it is crucial to also have engagement and congruency in environmentally responsible behaviors at the individual level, as well as having in place the policies, infrastructures and progress having already made at the country levels that could allow and further encourage pro-environmental behaviors and willingness to happen.

## Data Availability

The datasets analysed during the current study are available from the Leibniz-Institut für Sozialwissenschaften website at https://search.gesis.org/research_data/ZA7602.

## Footnotes

1 Sampling points were drawn systematically from each of the administrative regional units, after stratification by individual unit and type of area. Further information on instruments and sampling procedure used for each of the individual countries can be found in documentation at the ZACAT online data catalogue, or the Eurobarometer study descriptions in the data holding catalogue. At present, the dataset used for the study is the most recent available online, and can be accessed as ZA7602 data file version 1.0.0 from the *Leibniz-Institut für Sozialwissenschaften* website at https://search.gesis.org/research_data/ZA7602.
